# Gut microbiota and psoriasis: pathogenesis, targeted therapy, and future directions

**DOI:** 10.3389/fcimb.2024.1430586

**Published:** 2024-08-07

**Authors:** Xinyan Zou, Xinfu Zou, Longxia Gao, Hanqing Zhao

**Affiliations:** ^1^ College of Traditional Chinese Medicine, Hebei University, Baoding, Hebei, China; ^2^ First Clinical Medical College, Shandong University of Traditional Chinese Medicine, Jinan, Shandong, China

**Keywords:** psoriasis, gut microbiota, pathogenesis, targeted therapy, traditional Chinese medicine

## Abstract

**Background:**

Psoriasis is one of the most common autoimmune skin diseases. Increasing evidence shows that alterations in the diversity and function of microbiota can participate in the pathogenesis of psoriasis through various pathways and mechanisms.

**Objective:**

To review the connection between microbial changes and psoriasis, how microbial-targeted therapy can be used to treat psoriasis, as well as the potential of prebiotics, probiotics, synbiotics, fecal microbiota transplantation, diet, and Traditional Chinese Medicine as supplementary and adjunctive therapies.

**Methods:**

Literature related to the relationship between psoriasis and gut microbiota was searched in PubMed and CNKI.

**Results:**

Adjunct therapies such as dietary interventions, traditional Chinese medicine, and probiotics can enhance gut microbiota abundance and diversity in patients with psoriasis. These therapies stimulate immune mediators including IL-23, IL-17, IL-22, and modulate gamma interferon (IFN-γ) along with the NF-kB pathway, thereby suppressing the release of pro-inflammatory cytokines and ameliorating systemic inflammatory conditions.

**Conclusion:**

This article discusses the direction of future research and clinical treatment of psoriasis from the perspective of intestinal microbiota and the mechanism of traditional Chinese medicine, so as to provide clinicians with more comprehensive diagnosis and treatment options and bring greater hope to patients with psoriasis.

## 1 Introduction

Psoriasis is an inflammatory skin disease characterized by well-defined red patches covered with silvery-white scales (Boehncke and Schön, 2015). Psoriasis can manifest in various ways, often affecting the trunk, limbs, and joints, with plaque psoriasis being the most common type. When environmental and genetic factors activate plasmacytoid dendritic cells, cytokines such as TNF-α, IL-6, and IL-1β are released, leading to T cell-mediated inflammation, keratinocyte activation, and excessive proliferation, resulting in inflamed skin patches characteristic of psoriasis (Mahil et al., 2016). Inflammation not only affects the skin but also different organs throughout the body. Metabolic syndrome (Sommer et al., 2006; Gerdes et al., 2016),cardiovascular disease (Gelfand et al., 2006; Gelfand et al., 2009; Prodanovich et al., 2009; Ahlehoff et al., 2011), diabetes, depression, and other conditions are all associated with the severity of psoriasis.

The human gut microbiota is complex and diverse, consisting of bacteria, viruses, and fungi. Among them, bacteria are the most abundant, with over 99% of bacteria belonging to the phyla Firmicutes, Bacteroidetes, Actinobacteria, and Proteobacteria, with Firmicutes and Bacteroidetes dominating the gut microbiota of healthy individuals (Kostic et al., 2014). They play a crucial role in promoting nutrient absorption, preventing pathogen invasion, and regulating the immune system (Milani et al., 2017). A reduction in the relative abundance of microbiota and an increase in pathogenic bacteria can disrupt the homeostasis of gut microbiota composition and ecosystem, consequently influencing the body’s immune function and promoting the development of chronic inflammatory diseases (Stecher, 2015; Zhao et al., 2023).

Both the gut and the skin are dynamic and rich neuroendocrine organs with diverse microbiota. They maintain host internal balance through their respective physical, chemical barriers, and beneficial symbiotic microbial communities (Tlaskalová-Hogenová et al., 2004; Coates et al., 2019; Mahmud et al., 2022). Although the interaction mechanisms between gut microbiota and skin health are not yet fully understood, an increasing amount of research is beginning to explore how the gut microbiota, based on the gut-skin axis, influences the development of chronic inflammatory diseases like psoriasis and acne (Olejniczak-Staruch et al., 2021; Wang and Chi, 2021). This article employs the MESH thesaurus to accurately search literature on psoriasis and gut microbiota in PubMed. Simultaneously, it applies a keyword strategy in CNKI to delve deeper into relevant studies. The aim is to systematically explain the connection between microbial imbalance and psoriasis, as well as microbiome-targeted therapies for the prevention and treatment of psoriasis.

## 2 Intestinal microbiota composition and psoriasis

### 2.1 Intestinal bacterial dysbiosis and psoriasis

More and more research indicates that the gut microbiota of psoriasis patients has undergone changes in diversity and relative abundance of specific bacterial taxa compared to healthy individuals (Hidalgo-Cantabrana et al., 2019; Zhang et al., 2021). It has been reported that psoriasis patients show an increase in the abundance of Firmicutes and Actinobacteria, while the abundance of Bacteroidetes decreases (Chen et al., 2018; Shapiro et al., 2019). Furthermore, a study using 16S rRNA sequencing analysis of fecal microbiota in psoriasis patients found that at the genus level, Faecalibacterium and Megamonas were increased in abundance. Among these, Faecalibacterium prausnitzii (Zhang et al., 2021), whose supernatant has anti-inflammatory effects to maintain the healthy balance of the gut (Zhou et al., 2018). Meanwhile, genetic predictions suggest that Prevotella, Eubacterium, Lactobacillus, Odoribacter, and Slackia have significant causal effects on psoriasis (Mao et al., 2023). These findings suggest a close relationship between gut microbiota composition and psoriasis.

The ecological imbalance of the gut microbiota can promote immune reactions in the host’s intestinal mucosa, leading to the occurrence of systemic inflammatory diseases (Li et al., 2019). Related studies have shown that variations in the microbiota are associated with abnormal inflammatory markers in psoriasis patients. Specifically, in a study of gut microbiota and cytokines in fecal samples from patients with psoriasis, it was found that IL2R is positively correlated with Phascolarctobacterium and negatively correlated with Dialister (Zhang et al., 2021). Both Phascolarctobacterium and Dialister are involved in predicting the occurrence of inflammatory reactions and disease activity (Garshick et al., 2019; Zhang et al., 2021). Furthermore, the metabolic products of the gut microbiota, such as fatty acids, also influence intestinal mucosal health. Levels of medium-chain fatty acids (MCFAs), including caprylic acid and capric acid, were found to be significantly reduced in fecal samples from patients with psoriatic arthritis (PsA) and psoriasis (Ps) compared to healthy individuals. The antimicrobial properties of MCFAs are crucial for maintaining gut microbiota homeostasis (Scher et al., 2015). These reports emphasize the impact of the gut microbiota in the pathogenesis and progression of psoriasis (**Table 1**).

**Table 1:** Summary of the most relevant research studies on the gut microbiome of patients with psoriasis.

Author	Study Group	Controlling for confounding variables(BMI,Age,Gender)	Analysis Sample	Method of Analysis	Results
Yi-Ju Chen et al., 2018 (Chen et al., 2018)	Psoriasis patients (n = 32) Healthy controls (n = 64)	Yes	fecal samples	16S rRNA sequencing analyses(V3-V4 hypervariable region)	↓Bacteroidetes phylum, ↑Firmicutes phylum; ↓Bacteroidaceae family, Prevotellaceae family, ↑Ruminococcaceae family, Lachnospiraceae family
Codoñer FM et al., 2018 (Codoñer et al., 2018)	plaque psoriasis patients (n = 52) Healthy controls from Human Microbiome Project (n = 300)	No	fecal samples	16s rRNA sequencing (hypervariable region V3–V4)	↓genus Bacteroides ↑Akkermansia spp Ruminococcus
Tan et al., 2018 (Tan et al., 2018)	Psoriasis patients (n = 14) Healthy controls (n = 14)	No	fecal samples	16s rDNA sequencing (V4 hypervariable region)	↓Akkermansia muciniphila, Verrucomicrobia Tenericutes phyla Mollicutes Verrucomicrobiae ↑Bacteroides genera, Clostridium citroniae spp. Enterococcus genera
Hidalgo -Cantabrana et al., 2019 (Hidalgo-Cantabrana et al., 2019)	Psoriasis patients (n = 19) Healthy controls (n = 20)	No	fecal samples	16s rRNA sequencing (V2 -V3 hypervariable region)	↓ diversity ↑ Firmicutes ↓ Bacteroidetes ↑ F/B ratio ↑ Actinobacteria ↓ Proteobacteria phylum, Alistipes, Bacteroides, Barnesiella, Faecalibacterium, Parabacteroides and Paraprevotella genera
Jonathan Shapiro et al., 2019 (Shapiro et al., 2019)	Psoriasis patients (n = 24) Age-, BMI-, comorbidity-matched non-psoriasis controls (n = 22)	Yes	fecal samples	16S rRNA sequencing analyses(V4 hypervariable region)	↑Firmicutes phylum ↓Bacteroidetes phylum ↑F/B ratio ↑Blautia Genus Faecalibacterium Genus ↓Prevotella genus ↑Ruminoccocus gnavus Dorea formicigenerans Collinsella aerofaciens
Yegorov S et al., 2020 (Yegorov et al., 2020)	Psoriasis patients (n = 20) Healthy controls (n = 20)	Yes	fecal samples	16S rRNA gene sequencing	↑Lachnospiraceae family ↑Faecalibacterium ↓*Oscillibacter* *Roseburia*
Dei-Cas I et al., 2020 (Dei-Cas et al., 2020)	Psoriasis patients (n = 55) Healthy controls (n = 27)	Yes	fecal samples	16S rRNA sequencing analyses(hypervariable region V3–V4)	↑Firmicutes Proteobacteria Fusobacteria ↑*Faecalibacterium* *Blautia*
Valentini et al., 2021 (Valentini et al., 2021)	Psoriasis patients treated with biologic therapy (n = 10) Psoriasis patients not treated with biologic therapy (n = 20)	No	fecal samples	16s rRNA sequencing	↓ diversity of biologically treated patients vs. untreated patients
Xinyue Zhang et al., 2021 (Zhang et al., 2021)	Psoriasis patients (n = 30) Healthy controls (n = 30)	Yes	fecal samples	16S rRNA sequencing analyses	↑Veillonellaceae family Ruminococcaceae family ↓Lachnospiraceae genus ↑Faecalibacterium genus Megamonas genus prevotella genus
Wang et al., 2022 (Wang et al., 2022)	Severe Psoriasis patients (n = 28) Healthy controls (n = 21)	Yes	fecal samples	16S rRNA sequencing analyses	↓*Firmicutes* *Proteobacteria* ↑*Bacteroidetes* ↓unidentified_Enterobacteriaceae unidentified_Lachnospiraceae Romboutsia Subdoligranulum, unidentified_Erysipelotrichaceae, Dorea ↑Lactobacillus Dialister
Wen C et al., 2023 (Wen et al., 2023)	Psoriasis patients (n = 32) Healthy controls (n = 32)	Yes	fecal samples	16S rRNA gene amplicon sequencing approach	↑phylum Bacteroidetes ↓*Firmicutes* ↓Roseburia Eubacterium genus ↑Bacteroides ↓Roseburia hominis ↑ Escherichia spp Bacteroides uniformis

↓, decreased; ↑, increased; rRNA, ribosomal ribonucleic acid.

Furthermore, to minimize the influence of confounding factors such as diet and living environment on gut microbiota, rRNA sequencing analysis of gut microbiota was conducted in 17 patients with psoriasis and their healthy spouses (Wen et al., 2023). The results showed that, compared to healthy spouses, there were only differences at the species level; Alistipes finegoldii was increased and Bacteroides eggerthii was decreased in patients with psoriasis, with no differences observed at the phylum and genus levels. Significant differences in gene function were also found between patients with psoriasis and their healthy spouses. Therefore, we speculate that genes may regulate gut microbiota to some extent, resulting in differences in microbial abundance between patients and healthy individuals. Currently, there is relatively limited comprehensive research on the genetics and gut microbiota of patients with psoriasis. However, a study was conducted to analyze the gut microbiota, target gene pathways such as Kyoto Encyclopedia of Genes and Genomes(KEGG)and Clusters of Orthologous Groups(COG), and microbial metabolic functions in 30 patients with psoriasis (Xiao et al., 2021). It is revealed significant changes in the distribution of gut microbiota in patients with psoriasis compared to healthy controls. Additionally, they identified significant enrichment of 15 KEGG pathways, including lipopolysaccharide (LPS) biosynthesis, WNT signaling pathway, and apoptosis. Furthermore, five metabolites showed significant downregulation in the psoriasis cohort. These findings further underscore the complexity of psoriasis pathogenesis, influenced by multiple factors including the immune system, genetics, and gut microbiota.

### 2.2 The role of the gut microbiome in the pathogenesis of psoriasis

**Figure 1:**
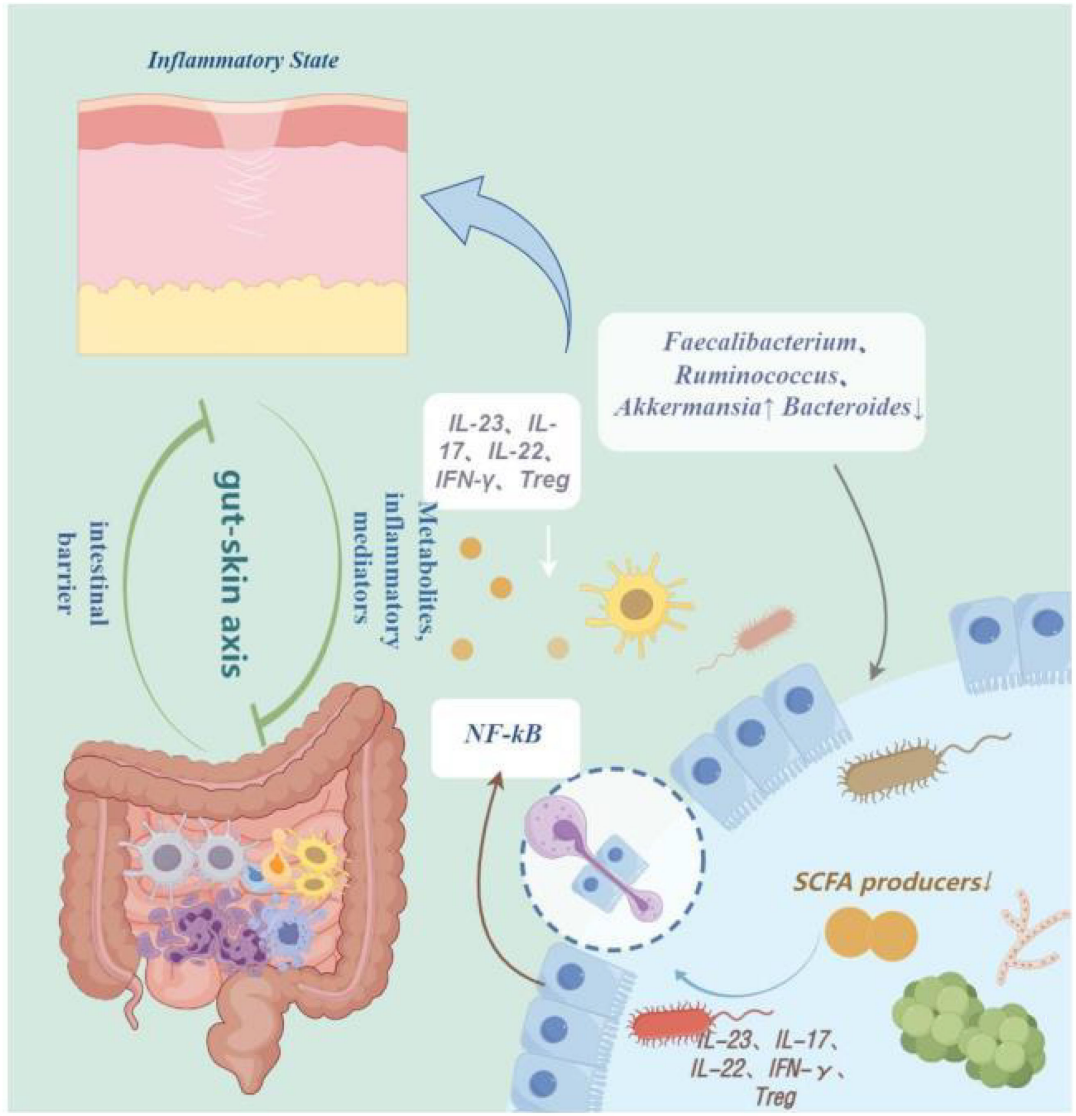
The pathogenesis of psoriasis. IL-23, Interleukin-23; IL-17, Interleukin-17; IL-22, Interleukin-22; IFN-γ, interferon-gamma; Treg, Regulatory T cells; NF-kB, Nuclear Factor-kappa B; NF-kB, Short-Chain Fatty Acids. By Figdraw.

The diversity and composition of the gut microbiota have a significant impact on the immune system and susceptibility to diseases, especially autoimmune diseases such as psoriasis (Kierasińska and Donskow-Łysoniewska, 2021). Studies have shown that in the intestines of psoriasis patients, there is a decrease in the phylum Bacteroidetes and an increase in the phylum Firmicutes, leading to changes in diversity (Komine, 2020). Additionally, multiple studies have shown that alterations in the gut microbiota composition participate in the pathophysiology of psoriasis by activating various immune mediators such as IL-23, IL-17, IL-22, regulating interferon-gamma (IFN-gamma), and inhibiting the production of T regulatory cells (Tregs) (Mann et al., 2020; Kapoor et al., 2022). Furthermore, large-scale genomic sequencing analysis based on 16S rRNA revealed changes in the gut bacterial composition of psoriasis patients, with an increase in Firmicutes, Akkermansia species, and Veillonella species, and a decrease in Bacteroidetes, leading to an imbalance in the gut microbiota that inhibits the production of short-chain fatty acids (SCFAs) - butyrate and propionate. This imbalance activates the NF-kB pathway, further activating inflammatory factors, triggering inflammation reactions, compromising barrier integrity, and participating in the pathogenesis of Psoriasis (Zheng et al., 2017; Codoñer et al., 2018; Stoeva et al., 2021; Valentini et al., 2021). Moreover, studies have found that the levels of Coprobacillus, Akkermansia, Veillonella, and Paraprevotella genera are decreased, which may exacerbate psoriasis symptoms due to the inhibition of autoimmunity by Streptomyces, reducing intestinal inflammation, and inducing Tregs. Thus, the lower the abundance of these genera, the more severe the symptoms of psoriasis may be (Visser et al., 2019; Sinha et al., 2021; Thye et al., 2022). It is evident that the gut microbiota plays a crucial role in maintaining host homeostasis and immune inflammatory responses. Furthermore, dysbiosis of gut microbiota may lead to excessive growth or abnormal increase of pathogens, thereby increasing the production and release of Pathogen-Associated Molecular Patterns(PAMPs) (Campbell et al., 2023; Kasarello et al., 2023). Despite limited reports on circulating PAMP levels in patients with psoriasis, existing studies have indicated that PAMPs or Damage-Associated Molecular Patterns(DAMPs) in psoriasis patients can be recognized by Pattern Recognition Receptors(PRRs, thereby activating immune responses in keratinocytes or Plasmacytoid Dendritic Cells(pDCs), promoting the release of various pro-inflammatory cytokines including IFNβ, IL1β, IL36, TNF, IL6, IL8, IL25, and CXCL10. These factors contribute to the formation of the inflammatory T cell phenotype in psoriasis (Albanesi et al., 2018; Xu et al., 2018; Sun et al., 2019). We speculate boldly that dysbiosis of gut microbiota causing PAMP release and subsequent immune activation may influence immune-mediated skin diseases such as psoriasis. While research has shown a certain association between the gut microbiota and psoriasis, the literature primarily focuses on microbial composition and immune inflammation, with limitations and a limited number of studies (Scher et al., 2015; Li et al., 2019; Sinha et al., 2021). Therefore, future exploration should broaden research directions to uncover other mechanisms of action between the gut microbiota and psoriasis, aiming to expand clinical approaches to diagnosis and treatment (**Figure 1**).

## 3 The microbial axis of gut-skin in psoriasis

An increasing number of studies indicate a close relationship between gut microbiota imbalance and various systemic inflammatory skin diseases, including psoriasis. Therefore, the concept of the gut-skin axis has been widely recognized and focused on in the medical community, linking skin diseases and microbial communities through metabolites, inflammatory mediators, and the intestinal barrier (Sikora et al., 2020). Although the mechanisms of interaction in the gut-skin axis have not been fully explored, gut microbiota seems to maintain skin homeostasis by regulating the systemic immune system (O’Neill et al., 2016). Microbial dysbiosis may trigger inflammatory reactions, leading to tissue or skin dysfunction (Salem et al., 2018; Polkowska-Pruszyńska et al., 2020). Numerous research results have found changes in the gut microbial balance in psoriasis patients, with an increase in the Bacteroides genus and a decrease in the Prevotella genus, similar results have been observed (Scher et al., 2015; Codoñer et al., 2018; Myers et al., 2019; Le et al., 2020). It is known that Prevotella activates regulatory T cells by producing polysaccharide A to play an immunomodulatory role in the intestine (Mosca et al., 2016). Therefore, a decrease in this genus may lead to changes in the immune response to the gut microbiota, promoting the upregulation of inflammatory factors IL-17 and TNF-α, further exacerbating the adverse effects related to the pathogenesis of psoriasis. Extensive animal experiments have also confirmed the preventive effect of Prevotella on inflammatory diseases by gavaging mice with this bacterium (Liu et al., 2022; Wu et al., 2023).

In addition, Okada Karin and others (Okada et al., 2020)analyzed the 16S rRNA gene of Staphylococcus aureus and Streptococcus pyogenes-rich gut microbiota in transgenic mice with keratinocyte-specific caspase-1 (Kcasp1Tg) inflammatory skin model. They then orally administered these dominant bacteria and control bacteria to wild-type mice treated with antibiotics, establishing a psoriasis-like skin inflammation model induced by imiquimod. It was observed that in the groups treated with Staphylococcus aureus and Streptococcus pyogenes, levels of inflammatory factors TNF-α, IL-17A, IL-17F, and IL-22 increased, exacerbating skin lesions. The results indicate that the severity of skin inflammation is associated with a vicious cycle between gut microbiota, where gut microbiota may act as both the cause and consequence of inflammation. Therefore, in addition to treating skin diseases, regulating gut microbiota may be an innovative approach for future treatment of inflammatory skin diseases such as psoriasis.

## 4 Interaction between gut microbiota and therapeutic drugs for psoriasis (Western medicine and traditional Chinese medicine)

### 4.1 Interaction between gut microbiota and Western medicine treatment for psoriasis

Psoriasis is characterized by abnormal activation of the immune system, particularly proliferation and activation of T cells and dendritic cells. In psoriasis patients, the skin is infiltrated by lymphocytes, macrophages, neutrophils, and abundant T cells, which are hallmark features of the disease. Specifically, Th1 and Th17 cells play crucial roles in the pathophysiology, releasing pro-inflammatory cytokines such as IL-17 and IFN-γ, accelerating keratinocyte proliferation, and triggering skin inflammatory responses. Furthermore, immune dysregulation may also lead to excessive proliferation of skin cells and abnormal keratin formation, further exacerbating the pathophysiological processes of psoriasis (Pasquali et al., 2019; Grän et al., 2020). Currently, the application of immunobiologics still dominates in the understanding of the pathogenesis of psoriasis. Drug interventions have significant impacts on the composition and function of gut microbiota. Medications interact directly or indirectly with gut microbiota through various pathways and cytokines, participating in bacterial metabolism. Clinical evidence suggests that biologics can alter microbial diversity (Bilal et al., 2018; Bai et al., 2019; Valentini et al., 2021). According to reports, the relative abundance of Roseburia, Lachnoclostridium, Bacteroides vulgatus, Anaerostipes, and Escherichia-Shigella increases with the administration of the IL-23 inhibitor (guselkumab); while the use of IL-17 inhibitors (secukinumab or ixekizumab) significantly increases the levels of Bacteroides stercoris and Parabacteroides merdae, and decreases the levels of Blautia and Roseburia. These changes in microbial diversity may modulate intestinal inflammatory responses (Shin et al., 2015; Yeh et al., 2019; Yao et al., 2022; Huang et al., 2023). Additionally, studies have shown that compared to untreated psoriasis, psoriasis patients successfully treated with ustekinumab exhibit increased proportions of Firmicutes, Pantoea, and unclassified_Comamonadaceae, but decreased proportions of Actinobacteria, Corynebacterium, Hydrogenophilaceae, and Streptococcus (Du et al., 2023). It is known that these bacteria can produce anti-inflammatory substances such as butyrate, medium-chain fatty acids (MCFAs) during bacterial metabolism, suppress intestinal oxidative stress, and regulate the balance between Th17/Treg lymphocytes. Therefore, changes in gut microbiota abundance are beneficial for improving symptoms of psoriasis (Kim et al., 2017; Clements and RC, 2018; Polak et al., 2021; Zhao et al., 2024). Apart from biologic agents, systemic treatments such as methotrexate and cyclosporine act through various mechanisms to modulate the immune system and reduce inflammatory responses, potentially influencing psoriasis treatment outcomes by directly or indirectly impacting the gut microbiota. Studies have shown that after treatment with methotrexate, patients who do not respond well tend to exhibit higher gut microbiota diversity and reduced metabolic pathways among gut microbes compared to those achieving favorable outcomes (Qiu et al., 2022). It is inferred that patients with higher gut microbiota diversity may develop resistance to methotrexate treatment. Given the scarcity of studies in this domain, these findings are preliminary and underscore the imperative for comprehensive investigations to substantiate the impact of systemic immunosuppressive therapies on the gut microbiota of psoriasis patients.

### 4.2 Interaction between gut microbiota and traditional Chinese medicine in the treatment of psoriasis

In China, traditional Chinese medicine has been widely used in the treatment of psoriasis, showing significant efficacy in improving the symptoms of psoriasis patients. However, the exact mechanism of action of traditional Chinese medicine is not fully understood and there are few reports on it. Recently, a large number of animal experiments have reported the mechanisms of single Chinese herbal medicine ingredients and compound formulas in improving the inflammatory mouse model of psoriasis (Nguyen et al., 2018; Zhang and Wei, 2020; Jin et al., 2021; Song et al., 2021; Tsiogkas et al., 2022). For example, It is found that rosin isolated from frankincense and administered by gavage to mice induced by imiquimod (IMQ) can improve inflammation by inhibiting cytokines associated with the IL-23/IL-17 immune axis, and conducted 16S rRNA sequencing analysis showing that rosin can reshape the gut microbiota of mice (Li et al., 2021). It has been reported that curcumin has been proven to reduce the production of pro-inflammatory factors such as IFN-γ and IL-17 in psoriasis patients to achieve anti-inflammatory effects (Skyvalidas et al., 2020). In addition, a study found that curcumin can induce changes in the gut microbiota and is closely related to inflammatory factors (Vollono et al., 2019). Specifically, Ligilactobacillus and Anaeroplasma are positively correlated with various psoriasis-related factors such as IL-6, IL-17A, IL-22, and IL-23, while Rikenella, Alistipes, and Mucispirillum are negatively correlated with them (Cai et al., 2023). Research has found that parthenolide can increase the abundance of gut microbiota Alloprevotella and Fournierella genera, thereby upregulating the immunosuppressive cytokine IL-10 in colonic tissue to alleviate systemic inflammatory responses (Liu et al., 2020). In summary, traditional Chinese medicine can improve the systemic inflammatory state of psoriasis patients by altering the abundance and composition of gut microbiota. However, since the composition of traditional Chinese medicine decoctions is complex, exploring the mechanisms of action of major components on gut microbiota and the interactions among them is a breakthrough for future research (**Table 2**).

**Table 2:** Summary of relevant research on traditional Chinese medicine treatment for psoriasis.

Author	Subject of Study	n1/n2 (E/C)	Experimental group intervention	Control group intervention and doses	Period of treatment	Outcome measure	Gut Microbiota Change	Other Outcome
Liet al., 2021 (Li et al., 2021)	Specific pathogen-free BALB/c male mice (7 weeks old, 16–18 g)	8/8	sodium abietate (40 mg/kg/d)	Imiquimod cream (5%) (62.5 mg/d)	7days	16S rRNA sequencing analyses(V3-V4 hypervariable region)	↑Bacteroidetes ↓*Firmicutes*, *Tenericutes* phylum ↑*Bacilli class* ↑*Bacillales order* ↑*Porphyromonadaceae* *Prevotellaceae, Planococcaceae* *Enterobacteriaceae family* ↓*Anaerotruncus Christensenella genus* ↑*Kurthia*, *Citrobacter*, and *Klebsiella genus*	↓the ratio of Th17 cells ↑Treg cell (CD4+/CD25+/Foxp3+) rate ↓IL-17A, IL-23, TNF-*α*, and IL-1*β*
Cai Z et al., 2023 (Cai et al., 2023)	BALB/c mice(8–10 weeks, 20–22 g)	4/4	curcumin (100 mg/kg/day)	imiquimod cream (62.5 mg/d)	6days	16S rRNA sequencing analyses(V3-V4 hypervariable region)	↑flora diversity ↑*Mucispirillum*, *unclassified_desulfovibrionaceae genus* ↑*Deferribacters*, *Desulfovibrionia class* ↑*clostridia, Deferribacteresw, and Desulfovibrionia class* ↑*Alistipes*, *Desulfovibrio*, *Mucispirillum*, and *Rikenella genus*	↑IL-6, IL-17A, IL-22, IL-23, TNF-α, TGF-β1, IL-10
Di T et al., 2021 (Di et al., 2021)	Babl/c male mice, aged 6–8 weeks,	8/8	Tuhuaiyin15 g/Kg intragastric administration	Imiquimod cream (5%) (62.5 mg/d)	7days	16S rRNA sequencing analyses	↑The diversity of intestinal microbiota ↑ ovatus, RF32, Christensenellaceae,Clostridium	↓IL-17A, IL-22, GRO ↑MCP-1, MIP-1 β
Huang Gang et al., 2023 (Huang Gang. et al., 2023)	BALB/c male mice (6–8 weeks, 20–22 g)	6/6	rhinoceros horn and rehmannia decoction 320mg/mL/d	Imiquimod cream (5%) (42 mg/d)	14days	Fecal bacterial culture	↑Lactobacillus, Bifidobacterium ↓Enterococcus, Escherichia coli	↓IL-17, IL-23,TNF-α
Huang Gang et al., 2022 (Huang et al., 2022)	Blood heat psoriasis	30/30	rhinoceros horn and rehmannia decoction 200ml,twice/d+Jinhuang Ointment	Acitretin Capsules 20mg/d+Jinhuang Ointment	8weeks	Fecal bacterial culture	↑Lactobacillus, Bifidobacterium ↓Enterococcus	↓Th17,Treg/Th17 ↑Treg
ZHANG Yuting et al., 2024 (Zhang et al., 2024)	Spleen dampness syndrome of psoriasis vulgaris	20/20	Spleen detoxification soup 250ml,twice/d+Calcipotriol Ointment	Healthy control	4weeks	16S rRNA sequencing analyses	↑Gammaproteobacteria,Actinobacteria, ↓Bacteroidota-Baacteroidia phylum ↑Enterobacteriaceae,Bifidobacteriacease family ↑Bifidobacterium Escherichia-Shigella ↓Bacteroides	
LUO Saijun.2022 (Luo, 2022)	BALB/c male mice (6 weeks, 20 g)	6/6	Zhuhuang granules250mg/ml	Imiquimod cream (5%) (42 mg/d)	7days	16S rRNA sequencing analyses	↑Alistipes,Lactobacillus,Muribaculaceae S24-7	↓IL-1*β*,IL-6,TNF-α
HU Hui-ying et al., 2020 (Hu et al., 2020)	Male SD mice	8/8	Runzao Zhiyang capsule(RZC)/d	5%Propranolol	2weeks	Fecal bacterial culture	↓Enterococcus, Escherichia coli ↑Lactobacillus. Bifidobacterium.	↓IL-6,TNF-α,IL-17 ↑IL-10

↓, decreased; ↑, increased; rRNA, ribosomal ribonucleic acid.

## 5 Targeted therapeutic applications of gut microbiota

### 5.1 Dietary intervention

Diet plays a direct role in the composition of the gut microbiota and the stability of the microbial ecosystem. There is evidence confirming a close relationship between dietary patterns, gut microbiota composition, and psoriasis. The Mediterranean diet (MD) emphasizes the intake of fruits, vegetables rich in polyphenols, fiber, and vitamins, as well as fish, seafood, and nuts high in polyunsaturated fatty acids, which is recognized as a model of healthy eating (Gantenbein and Kanaka-Gantenbein, 2021; García-Montero et al., 2021; Kiani et al., 2022). The antioxidant and anti-inflammatory properties of polyphenols, polyunsaturated fatty acids, dietary fiber, and vitamins have been well established (Liu et al., 2019 2019; Liu et al., 2021; Zhang et al., 2021; Harwood, 2023; Kong et al., 2023; Tian et al., 2023). Clinical studies have shown that intervention with the MD can help inhibit the progression of psoriasis, enrich the diversity of gut microbiota, indicating an intervention effect of the MD on psoriasis (Lubberts et al., 2022; Cintoni et al., 2023). A cross-sectional observational study of psoriasis patients found that the Mediterranean diet can improve symptoms of psoriasis and is negatively correlated with the severity of psoriasis (Phan et al., 2018; Zanesco et al., 2022). The MD can alter the diversity of gut microbiota, influence inflammatory markers, participate in intestinal inflammatory responses, and serve as a major regulator between gut microbiota and the immune system (Fiorindi et al., 2021; Sugihara and Kamada, 2021; Yan et al., 2022). Research indicates that the MD can increase the levels of Bifidobacterium, Actinobacteria, and bacteria producing short-chain fatty acids (Clostridium leptum and Faecalibacterium), decrease levels of Helicobacter pylori, Firmicutes, and Cyanobacteria, regulate the ratio of beneficial to harmful bacteria, reduce inflammation and oxidative reactions, enhance intestinal immunity, and alleviate symptoms of psoriasis (Garbicz et al., 2021; Barber et al., 2023). An experimental study by Takahashi et al. found that fucoidan increased the levels of Desulfovibrionaceae, Actinobacteria, Enterococci, and Desulfobacter, decreased levels of Lachnospiraceae and Ruminococcaceae, increased short-chain fatty acids, and interacted with non-Toll-like pattern recognition receptors such as dectin-1 and complement receptor-3 to activate macrophages, neutrophils, and helper T cells to stimulate innate immunity and improve the course of psoriasis (Sawin et al., 2015; Sun et al., 2017; Garbicz et al., 2021). In conclusion, based on the gut microbiota, dietary therapy as a treatment approach for psoriasis is worth further promotion. Developing a comprehensive and stable dietary plan is beneficial for achieving optimal therapeutic outcomes.

### 5.2 Bioactive dietary components

#### 5.2.1 Omega-3 fatty acids

Dietary omega-3 polyunsaturated fatty acids can inhibit intestinal inflammation, maintain intestinal homeostasis, alter gut microbiota diversity, and enhance host immune function (Calder, 2017; Bellenger et al., 2019). It has been reported that fish oil, rich in omega-3 fatty acids, can inhibit Escherichia coli while increasing the levels of Bifidobacteria, thereby reducing lipopolysaccharide-related inflammatory responses (Cao et al., 2019). Furthermore, studies have shown that administering flaxseed oil to rats can increase the production of short-chain fatty acids (SCFAs) and gut microbiota diversity, with a negative correlation observed between lactic acid bacteria, Firmicutes, Bacteroidetes, and Bifidobacteria and pro-inflammatory markers (IL-1β, IL-6, IL-10, IL-17A, and TNF-α) (Wang et al., 2020). This may explain the involvement of omega-3 fatty acids in the pathogenesis of psoriasis. Research on a mouse model of psoriasis induced by imiquimod (IMQ) and intervened with Resolvin E1 (RvE1), a metabolite of omega-3 polyunsaturated fatty acids, has shown that RvE1 can terminate the inflammatory process by inhibiting Leukotriene B4 - BLT1(LTB4-BLT1) signaling and regulating the expression of Th17 and Tc17 cytokines, effectively inhibiting inflammatory cell infiltration and epidermal hyperplasia in psoriatic skin, thereby improving the severity of psoriasis (Sawada et al., 2018; Oner et al., 2021). Additionally, clinical studies have demonstrated that sustained intake of omega-3 fatty acids significantly increases the abundance of Coprococcus, Bacteroides, and Bifidobacteria in the gut, while decreasing the relative abundance of bacteria that produce SCFAs (such as clostridia and certain types of rumen cocci) (Vijay et al., 2021). Coprococcus has been implicated in the physiological and pathological processes of psoriasis (Sun et al., 2021; Yu et al., 2023),while the anti-inflammatory and immunomodulatory mechanisms of SCFAs are well recognized (Fusco et al., 2023; Kim, 2023; Zhang et al., 2023). Moreover, omega-3 polyunsaturated fatty acids can inhibit the binding of Toll-like receptor-4 (TLR4), thereby suppressing the expression of the NF-kB pathway and the secretion of pro-inflammatory cytokines, similar to the pathogenesis of psoriasis (Arjomand Fard et al., 2023). Therefore, the application of omega-3 fatty acids can modulate gut microbiota composition and homeostasis, regulate inflammatory responses, participate in the pathophysiological processes of psoriasis, and improve symptoms in patients with psoriasis.

#### 5.2.2 Resveratrol

Resveratrol is a natural polyphenol compound, which is widely present in substances such as grapes, wine, and peanuts (Wang et al., 2022; Shahcheraghi et al., 2023; Wang et al., 2024). It has rich biological activities, such as anti-inflammatory, anti-oxidative stress, and immune regulation (Brito Sampaio et al., 2022; Coutinho-Wolino et al., 2022; Hsu et al., 2023; Eduardo Iglesias-Aguirre et al., 2023; Yang et al., 2023). Research has shown that resveratrol can exert anti-inflammatory effects by down-regulating toll-like receptor 4 in the intestines, reducing the gene expression of pro-inflammatory cytokines IL-1β, IL-6, and MMPs, as well as inhibiting the activity of innate immune markers TLR-2 and TLR-4 (Grosu et al., 2020; Pistol et al., 2021). At the same time, numerous studies have indicated that resveratrol can regulate the diversity of intestinal microbiota, inhibit intestinal inflammation, and protect intestinal barrier (Zhao et al., 2018; Chen et al., 2023). Resveratrol therapy has been shown to increase the abundance of Actinomycetes, Atopobiaceae, and Lactobacillus genera, while regulating the diversity of intestinal microbiota, improving intestinal function (Yao et al., 2022; Gao et al., 2023). Furthermore, an animal study demonstrated that the application of resveratrol can reduce harmful bacteria such as Desulfovibrio, Lachnospiraceae_NK4A316_group, and Alistipes, and increase the abundance of bacteria producing short-chain fatty acids (SCFA) like Allobaculum, Bacteroides, and Blautia in mice, thereby restoring intestinal mucosal morphology and improving intestinal barrier integrity (Li et al., 2020). As mentioned earlier, the ratio of Firmicutes/Bacteroidetes is often lower in patients with psoriasis (Liu et al., 2024); therefore, resveratrol can play a therapeutic role in psoriasis patients by acting on the intestinal microbiota (Marko and Pawliczak, 2023).

#### 5.2.3 Quercetin

Quercetin is a special subclass of flavonoids, which belongs to the polyphenolic compound category (Singh et al., 2021).Quercetin can be found in many fruits, Chinese herbs, vegetables, and plants (Ashrafizadeh et al., 2021; Alizadeh and Ebrahimzadeh, 2022). Its anti-inflammatory, antioxidant stress, and antibacterial properties have been widely recognized and verified (Li et al., 2018; Hosseini et al., 2021; Georgiou et al., 2023). In recent years, with the introduction of the concept of gut microbiota, many research directions have begun to converge towards this area, and quercetin also has a significant impact on gut microbiota (Santangelo et al., 2019; Kasahara et al., 2023; Roshanravan et al., 2023). Studies have shown that supplementing quercetin to pigeons induced by LPS can increase the relative abundance of some bacteria that produce short-chain fatty acids (SCFA) or promote health, such as Phascolarctobacterium, Negativicutes, Selenomonadales, Megamonas, Prevotellaceae, and Bacteroides_salanitronis, enhance the diversity of the gut microbiota to maintain intestinal health and participate in enhancing intestinal immunity (Feng et al., 2023). Furthermore, an *in vitro* study showed that quercetin can significantly increase the relative abundance of probiotics in the gut, with the most significant increase in Bifidobacterium, which can reduce the relative abundance of Clostridium difficile and Escherichia coli to regulate the composition of gut microbiota (Pan et al., 2023). In addition, quercetin increases the relative abundance of Bacteroides, Akkermansia, Butyricicoccus, Faecalibacterium, and Coprobacillus, while reducing the relative abundance of Proteobacteria, increasing antioxidant capacity to reduce intestinal damage (Xu et al., 2021). An animal experiment studying the improvement of psoriasis symptoms by quercetin supplementation showed that quercetin can significantly reduce the levels of TNF-α, IL-6, and IL-17 induced by imiquimod in mouse serum, simultaneously inhibit NF-κB signaling activation, enhance anti-inflammatory and antioxidant properties, thereby reducing psoriasis severity index (PASI) scores and improving symptoms (Chen et al., 2017). In addition, intervention with quercetin [main component of ethanolic extract (ESW)] showed less evident hyperkeratosis and cell infiltration compared to the control group, significantly improving symptoms such as erythema, scaling, and skin thickness of psoriasis, which may be related to the elevated levels of histidine, as branched-chain amino acids effectively reduce oxidative and inflammatory responses, playing a crucial role in regulating immune cell function (Li et al., 2021; Quante et al., 2022; Sharma et al., 2024). Although existing literature indirectly proves the certain connection between quercetin, gut microbiota, and psoriasis, there are relatively few corresponding experimental studies, which should be focused on in future research, especially the regulatory effects of quercetin on the gut microbiota of psoriasis patients (**Table 3**).

**Table 3:** The effects of biologically active compounds on the intestinal microbiome.

Author	Subject of Study	biologically active compounds	Intervention	Outcomes
Ting Wang et al., 2020 (Wang et al., 2020)	Female SD rats (6weeks, 193 ± 10g	Omega-3 fatty acids	1mg/kg/day of flaxseed oil by gavage for 8 weeks	↑Allobaculum, ↑Lactobacillus, ↑Butyrivibrio, ↑Desulfovibrio, ↑Bifidobacterium, ↑Faecalibacterium, ↑Parabacteroides ↓Actinobacteria, ↓Bacteroides, ↓Proteobacteria, ↓Streptococcus, ↓Firmicutes/Bacteroidetes ratio
Kåre Steinar Tveit et al., 2020 (Tveit et al., 2020)	Psoriasis patients n = 64. Randomized PASI < 10 53% of subjects used local anti-psoriatic maintenance treatment	Omega-3 fatty acids	Herring roe oil (containing 292 mg of polyunsaturated fatty acids omega-3), Daily dose: 2,6 g EPA and DHA	↓PASI score No difference in inflammatory markers
Amrita Vijay et al., 2021 (Vijay et al., 2021)	Healthy subjects n = 69 Randomized No previous treatment	Omega-3 fatty acids	Daily dose of 500 mg of omega 3 (165 mg EPA, 110 mg DHA)	↑iso-valerate ↑iso-butyrate ↑ Coprococcus ↑ Bacteroides ↓Colinsella.
Ting-Ting Cai et al., 2020 (Cai et al., 2020)	Male C57BL/KsJ diabetic db/db mice	Resveratrol	Oral administration of 10 mg/kg/day resveratrol for 12 weeks	↑Bacteroides, ↑Alistipes, ↑Rikenella, ↑Odoribacter, ↑Parabacteroides, ↑Alloprevotella
Pan Wang et al., 2020 (Wang et al., 2020)	High-fat diet-fed mice	Resveratrol	300 mg/kg/day resveratrol for 16 weeks	↑Lachnospiraceae family
Alharris E et al., 2022 (Alharris et al., 2022)	Female BALB/c mice(6-8weeks)	Resveratrol	200µl carboxymethyl cellulose (CMC) solution containing RES (100mg/kg) for 2 weeks	↑ Bacteroidetes ↓Firmicutes ↑Bacteroidales order,*Bacteroides acidifaciens* species ↑ SCFA, butyric acid,
Li Z et al., 2022 (Li et al., 2022)	Adult Sprague Dawley Intrahepatic cholestasis of pregnancy (ICP) rats	Resveratrol	oral administration of resveratrol (RSV)(60 mg/(kg/d) (750 mg RSV+2 ml DMSO+98 ml NS))	↓the richness and diversity of gut bacteria ↑Actinobacteria, Synergistetes, and Chloroflexi; Resveratrol can reverse the increase of Ruminiclostridium and Bilophila and the decrease of Actinobacteria
Lijun Zhao et al., 2021 (Zhao et al., 2021)	Monosodium glutamate-induced abdominal obese mice	Quercetin	5 mg/kg quercetin dissolved in 0.15% carboxymethylcellulose sodium, administrated by gavage for 6 weeks	↓Firmicutes/Bacteroidetes ratio ↓Firmicutes ↓Bacteroides spp. ↓Lachnospiraceae spp., ↓Ruminicoccaceae spp.
Su L et al., 2022 (Su et al., 2022).	C57BL/6J mice (male, four weeks old, weight 20–22 g)	Quercetin	quercetin (95% purity)(50 mg per kg BW per day)	improved intestinal epithelial barrier damage, and reduced intestinal permeability; ↑Firmicutes/Bacteroidetes ratio ↑Coprococcus_1, Akkermansia, Lactococcus, Allobaculum ↓Adlercreutzia

↓—decreased; ↑—increased;.

### 5.3 FMT

Fecal Microbiota Transplantation (FMT) is a method of transferring feces from a healthy donor to the colon of a patient, directly altering the recipient’s gut microbiota to normalize its composition and obtain therapeutic benefits (Vindigni and Surawicz, 2017; Wang et al., 2019). In various animal studies, it has been well established that transferring gut microbiota from healthy individuals can prevent psoriasis-like skin inflammation (Chen et al., 2021; Zhao et al., 2023). Analysis of Th17 and Treg cells in the spleen of mice revealed a significant decrease in the frequency and count of Th17 cells, as well as a significant increase in the transcription levels of the anti-inflammatory factor IL10 in FMT mice from healthy individuals, which is related to the alleviation of skin inflammation (Chen et al., 2021). Furthermore, multiple studies have reported improvements in psoriatic arthritis patients after FMT (Kragsnaes et al., 2018; Kragsnaes et al., 2024), with significant changes observed in plasma levels of TNF, IFN-γ, and Signaling Lymphocytic Activation Molecule Family Member 1(SLAMF1). A case report from China showed that after two FMT treatments, the skin lesions of a patient with plaque psoriasis basically disappeared, the affected body surface area (BSA) decreased to 6%, and the concurrent irritable bowel syndrome (IBS) significantly improved, with serum TNFα dropping to 13.7 ng/L, a decrease of 88.6%. This provides a new potential treatment for the disease (Yin et al., 2019). Despite studies confirming the safety of FMT in treating psoriasis (Kragsnaes et al., 2021), there are various issues in its clinical application, such as the risk of infections during the transplant process, recipient immune rejection of the donor, adverse reactions like nausea, vomiting, and diarrhea, and the impact of dietary and lifestyle habits on the composition of gut microbiota. These are challenges that need to be explored and addressed.

### 5.4 Probiotics/prebiotics/synbiotics/postbiotics

Probiotics are living microorganisms, including bacteria and yeasts, which have a positive impact on health by influencing the resident gut microbiota, intestinal barrier, and systemic immune system (Wieërs et al., 2019). Currently, the most commonly used probiotics include certain strains of Lactobacillus, Bifidobacterium, and Enterococcus (Sánchez et al., 2017). Numerous animal studies have shown that introducing Bifidobacterium or Lactobacillus strains into the gut environment of mice has a significant effect on the composition of the gut microbiota (Arthur et al., 2013; Turroni et al., 2016). Furthermore, the connection between probiotics and the immune system is a recent research focus. Probiotics can influence inflammatory responses and immune balance by directly or indirectly affecting the signaling pathways of the immune system. Specifically, probiotics can decrease Th17 polarization and shift T cells towards the Treg subset through membrane receptors, resulting in high levels of IL-10 and low levels of TNF-α, thereby reducing inflammation. On the other hand, probiotics can promote dendritic cells (DCs) to produce cytokines (such as IL-12 and IL-15) to stimulate the activation of natural killer (NK) cells (Maldonado Galdeano et al., 2019; Yousefi et al., 2019; Zhang et al., 2019; Cristofori et al., 2021; Potrykus et al., 2021). In various clinical trials, probiotics have been shown to significantly improve the severity of skin lesions and quality of life in psoriasis patients, and reduce levels of inflammatory markers such as high-sensitivity C-reactive protein(hs-CRP) and IL-6 (Moludi et al., 2021; Moludi et al., 2022; Choy et al., 2023).

Prebiotics are naturally present in carbohydrates in the human diet, which not only promote the growth of beneficial probiotic microorganisms in the human intestinal tract but also stimulate the immune system. Common prebiotics include inulin, oligofructose, oligogalactose, etc. (Markowiak and Śliżewska, 2017; Yadav et al., 2022) Studies have found that an important mechanism by which prebiotics affect the immune system is by altering the expression of cytokines. Specifically, a complex of inulin and oligofructose can significantly reduce the expression of the pro-inflammatory cytokine IL-1β in the cecum (Hoentjen et al., 2005), and it has been found that elderly people consuming galactooligosaccharides (5.5g/day) for 10 weeks can increase the production of the anti-inflammatory cytokine IL-10, while reducing the production of pro-inflammatory cytokines IL-1, IL-6, and TNF-α (Vulevic et al., 2008; Shokryazdan et al., 2017; Peters et al., 2019; Samanta, 2022). Mihaela Cristina Buhaş (Buhaş et al., 2023) and colleagues observed the effectiveness of probiotics combined with prebiotics in the treatment of psoriasis patients, and found that after 12 weeks of intervention, inflammatory markers TNF-α, IL-6, IFN-γ in psoriasis patients significantly decreased, and the main inhibitory factor of the immune response IL-10 increased. In addition, a decrease in triglyceride levels was observed, which is consistent with previous reports (Ooi and Liong, 2010; Bock et al., 2021).

Synbiotics are a synergistic combination of probiotics with added prebiotics. They can effectively alter the composition of the gut microbiota and enhance the epithelial barrier (Li et al., 2020; Nguyen et al., 2022). Studies have shown that continuous intake of synbiotics for three months can significantly increase the abundance of Bifidobacteria, Lactobacilli, as well as Cyanobacteria, Archaea, Clostridia, and Bacilli in the intestine, all of which are beneficial for gut health (Sergeev et al., 2020; Álvarez-Arraño and Martín-Peláez, 2021). In addition, Ali Akbarzadeh and colleagues found that synbiotics can significantly increase the levels of Fe, Zn, P, Mg, Ca, and Na in the serum of patients with mild to moderate psoriasis. The increase or decrease of certain trace elements and oxidative stress status have an impact on the development of psoriasis (Akbarzadeh et al., 2022). In summary, probiotics, prebiotics, and synbiotics have significant effects on altering the gut microbiota, stimulating the immune system, and increasing mineral absorption.

Postbiotics are biologically active compounds or metabolites produced after probiotics undergo fermentation or interact with gut microbiota. Among them, short-chain fatty acids and peptides are common postbiotics (Zhou et al., 2024). Research has shown that postbiotics can modulate the immune system by directly interacting with immune cells in gut-associated lymphoid tissue (GALT). Specifically, short-chain fatty acids (SCFAs) and polysaccharide peptides can inhibit inflammatory cytokines such as IL-1β, IL-6, IL-8, and TNF-α, induce the expression of anti-inflammatory cytokine IL-10, and promote the formation of regulatory T cells (Tregs) (Nakkarach et al., 2021; Yuan et al., 2023). A study involving 52 patients with psoriasis, where after 8 weeks of treatment with the E3 probiotic formula containing prebiotics, probiotics, and postbiotics orally, significant improvements were observed in Psoriasis Area and Severity Index (p < 0.001), as well as in Dermatology Life Quality Index (p = 0.009), with no adverse reactions reported. Additionally, the study found that gut microbiota in healthy controls may be more inclined towards metabolic pathways associated with short-chain fatty acids, whereas in the psoriasis group, functional abundance related to short-chain fatty acids (SCFA) was significantly reduced (Choy et al., 2023).

## 6 The impact of gut microbiota on other skin disorders

The composition of human microbiota is complex. These microbiota collectively maintain skin homeostasis by regulating immune cells present in the gut and skin. However, overgrowth and changes in diversity of gut microbiota can lead to the development of skin disorders. Imbalance in gut microbiota can result in three common skin disorders: psoriasis, atopic dermatitis, and acne.

Several studies on the role of gut microbiota in the pathogenesis of infantile atopic dermatitis (AD) suggest that infants with limited gut microbial diversity may develop AD later in life. It has been found that infants with IgE-related eczema have lower diversity of Actinobacteria and Bacteroidetes compared to healthy infants (Abrahamsson et al., 2012). Furthermore, plasma levels of short-chain fatty acids (SCFAs) are significantly reduced in four-month-old infants with atopic dermatitis (Barman et al., 2024). Additionally, diet may also influence the occurrence of AD. For example, a gluten-containing diet can lead to villus atrophy, intestinal inflammation, and gluten sensitivity is closely related to gut microbiota (de Sousa Moraes et al., 2014; Lorenzo Pisarello et al., 2015; Marasco et al., 2020; Mahmud et al., 2022). However, the causal relationship between gut microbiota and atopic dermatitis has not been confirmed. The pathogenesis of atopic dermatitis is mainly attributed to the imbalance of Th1 and Th2. Activated dendritic cells (DCs) migrate to local lymph nodes to induce naive T helper cells and polarize them into a Th2 phenotype. Subsequently, Th2 cells are recruited back to the skin and, along with the effector cytokines of Th22 cells, collectively induce skin inflammation (Biedermann et al., 2015).

Acne is a chronic inflammation of the pilosebaceous unit, closely related to excessive sebum production, follicular hyperkeratosis, and proliferation of Propionibacterium acnes (Clark et al., 2017). Most importantly, changes in the gut microbiota have been observed in acne patients. It has been reported that in acne patients, there is a lower abundance of Firmicutes but a higher abundance of Actinobacteria (Deng et al., 2018). Furthermore, there are gender differences in the gut microbiota of patients with acne vulgaris. Specifically, in the intestinal tract of males, there is a reduction in Cutibacterium acnes, Clostridium spp., and fecal bacteria, while in female patients, there is an increase in Clostridium perfringens and a decrease in Oscillibacter and Odoribacter (Huang et al., 2021). It has been reported that the gut microbiota of patients with moderate to severe acne shows a decrease in Actinobacteria and an increase in Bacteroidetes (Yan et al., 2018; Siddiqui et al., 2022). According to recent Mendelian randomization results, lactobacilli have a protective effect on acne, primarily through inhibition of the mTOR pathway and activation of the AMP-activated protein kinase pathway(AMPK) pathway, acting as protective factors (Monfrecola et al., 2016; Ji et al., 2024).

## 7 Summary and prospects

In recent years, regulating the gut microbiota to improve symptoms of diseases has become a key research focus. Although literature has confirmed a certain connection between gut microbiota and psoriasis, this area still faces many challenging problems and obstacles in future development. Here are some constructive suggestions for reference:

Delve deeper into and explore the impact of changes in gut microbiota on psoriasis from different perspectives. Current literature shows a lack of experimental research exploring the relationship between gut microbiota and psoriasis, often due to insufficient sample sizes. Additionally, the studies included in this review solely encompass adult populations. Therefore, in future research to elucidate the causal relationship between gut microbiota, psoriasis, and the pathogenesis, not only is a large amount of animal experimental data needed, but human experiments are also crucial to ensure the reliability and stability of experimental results. Through various microbiota-targeted therapies, adjusting the gut microbiota diversity of psoriatic patients to improve their symptoms could provide clinicians with broader diagnostic and therapeutic strategies.

Many studies have discussed the importance of the gut-brain-skin axis in neurologic diseases and gut microbiota, so investigating how the microbiota-gut-skin axis functions in the relationship between gut microbiota and psoriasis is essential.

As the composition of gut microbiota and the ratios of certain bacterial species are closely related to the severity and status of psoriasis, in the future, comprehensive evaluations of the severity of psoriatic symptoms, nutritional indices, gut microbiota species, and quantities are prerequisites for microbiota-targeted therapy application. This can provide patients with more refined treatment plans to better alleviate their suffering.

Prebiotics, probiotics, synbiotics, fecal microbiota transplantation, dietary microbiota-targeted therapy, as well as external treatment methods in traditional Chinese medicine (e.g. acupuncture), can maintain dynamic balance of the gut microbiota ecosystem from a microscopic perspective through various pathways and mechanisms, regulate the host’s immune system, improve the symptoms of psoriasis, and maintain overall health of the body. As these novel treatment methods are perfected and promoted, they will inject new strength into the clinical diagnosis and treatment of psoriasis.

## 8 Unresolved issues

Although current research and literature have demonstrated the inseparable connection between psoriasis and the diversity of gut microbiota and stability of the ecosystem, there are still many unresolved issues in the existing literature: (1) What are the differences in gut microbiota composition corresponding to different severity levels of psoriasis? (2) Besides gut immune reactions, are there other pathogenic mechanisms in the gut microbiota ecosystem? (3) When comparing studies, improvements are needed in the collection, transportation, storage, and DNA extraction protocols in both animal and human studies. Traditional Chinese medicine has shown significant efficacy in improving the symptoms of psoriasis, but it is worth contemplating and exploring how traditional Chinese medicine and gut microbiota therapy can be combined and whether new symptoms may arise after combination, in order to provide more choices for clinical treatment.

## Author contributions

XYZ: Methodology, Writing – original draft. XFZ: Data curation, Writing – original draft. LG: Supervision, Writing – review & editing. HZ: Writing – review & editing, Formal Analysis, Supervision.
